# Supplementary material for: The Depression Anxiety Stress Scale 21: Development and Validation of the Depression Anxiety Stress Scale 8-Item in Psychiatric Patients and the General Public for Easier Mental Health Measurement in a Post COVID-19 World
Source: Int J Environ Res Public Health. 2021 Sep 27;18(19):10142. doi: 10.3390/ijerph181910142 (PMC8507889; doi:10.3390/ijerph181910142)
Supplement: Supplementary file 1 [file ijerph-18-10142-s001.zip › Supplementary Table S1.pdf]

Supplementary Table S1. Invariance of factor structures of the shortened versions of the Depression Anxiety Stress Scale 21 across gender groups

Model	Samples	Invariance levels	χ^2	df	*p*	$\Delta\chi^2$	Δdf	$p(\Delta\chi^2)$	CFI	ΔCFI	TLI	ΔTLI	RMSEA	ΔRMSEA	SRMR
Osman 3F	Sample 1	Configural	84.952	46	0.000				.942		.909		.071		0.0420
9-item		Metric	99.227	52	0.000	14.275	6	0.027	.929	0.013	.902	0.007	.074	-0.003	0.0439
DASS		Strong	102.678	58	0.000	3.451	6	0.751	.933	-0.004	.917	-0.015	.068	0.006	0.0464
		Strict	113.964	68	0.000	11.286	10	0.336	.931	0.002	.927	-0.010	.064	0.004	0.0482
Osman 3F	Sample 2	Configural	119.758	44	0.000				.972		.955		0.042		0.0337
9-item		Metric	125.144	50	0.000	5.385	6	0.495	.973	-0.001	.961	-0.006	.039	0.003	0.0343
DASS		Strong	133.224	56	0.000	8.080	6	0.232	.972	0.001	.964	-0.003	.037	0.002	0.0363
		Strict	205.077	67	0.000	71.933	11	0.000	.950	**0.022**	.946	0.018	.046	0.009	0.0362
3F 12-item	Sample 1	Configural	155.932	98	0.000				.947		.929		0.060		0.0425
DASS		Metric	168.593	107	0.000	12.662	9	0.179	.944	0.003	.931	-0.002	.059	0.001	0.0439
		Strong	175.947	113	0.000	7.354	6	0.289	.943	0.001	.933	-0.002	.058	0.001	0.0457
		Strict	190.934	127	0.000	14.987	14	0.379	.942	0.001	.940	-0.007	.055	0.003	0.0467
3F 12-item	Sample 2	Configural	318.927	98	0.000				0.953		0.937		0.048		0.0398
DASS		Metric	331.824	107	0.000	12.897	9	0.167	.952	0.001	.941	-0.004	.046	0.002	0.0406
		Strong	358.693	113	0.000	26.868	6	0.000	.948	0.003	.939	0.002	.047	-0.001	0.0459
		Strict	465.739	127	0.000	107.046	14	0.000	.928	**0.020**	.925	0.014	.052	-0.005	0.0409
3F 13-item	Sample 1	Configural	213.586	124	0.000				.930		0.912		.066		0.0494
DASS		Metric	226.997	134	0.000	13.412	10	0.202	.927	0.003	.915	-0.003	.065	0.001	0.0483
		Strong	229.309	140	0.000	2.312	6	0.889	.930	-0.003	.922	-0.007	.062	0.003	0.0487
		Strict	248.062	153	0.000	18.753	13	0.131	.925	0.005	.924	-0.002	.061	0.001	0.0493

3F 13-item	Sample 2	Configural	318.927	98	0.000				0.953		0.937		0.048		0.0398
DASS		Metric	331.824	107	0.000	12.897	9	0.167	.952	0.001	.941	-0.004	0.046	0.002	0.0406
		Strong	358.693	113	0.000	26.868	6	**0.000**	.948	0.004	.939	0.002	0.047	-0.001	0.0459
		Strict	465.739	127	0.000	107.046	14	0.**000**	.928	**0.020**	.925	0.014	0.052	-0.005	0.0409
Our 3F 9-	Sample 1	Configural	62.697	46	.051				.985		.977		.047		0.0270
item DASS		Metric	63.249	52	.136	0.552	6	0.997	.990		.986		.036		0.0272
		Strong	65.920	58	.222	2.671	6	0.849	.993		.991		.029		0.0285
		Strict	81.410	68	.128	15.490	10	0.115	.988		.987		.034		0.0301
Our 3F 9-	Sample 2	Configural	171.666	46	0.000				.972		.956		.053		0.0252
item DASS		Metric	180.024	52	0.000	8.357	6	0.213	.972	0.000	.961	-0.005	.050	0.003	0.0255
		Strong	210.288	58	0.000	30.264	6	0.000	.966	0.006	.958	0.003	.051	-0.001	0.0333
		Strict	278.707	68	0.000	68.419	10	0.000	.953	0.013	.951	0.007	.056	-0.005	0.0257
Our 3F 8-	Sample 1	Configural	50.138	32	0.022				.982		.968		.058		0.0234
item DASS		Metric	50.572	37	0.068	0.434	5	0.994	.987	-0.005	.980	-0.012	.047	0.011	0.0239
		Strong	53.959	43	0.122	3.387	6	0.759	.989	-0.002	.986	-0.006	.039	0.008	0.0257
		Strict	68.265	52	0.065	14.306	9	0.112	.984	0.005	.983	0.003	.043	-0.004	0.0284
Our 3F 8-	Sample 2	Configural	92.271	30	0.000				.985		.973		.046		0.0206
item DASS		Metric	101.181	35	0.000	8.910	5	0.113	.985	0.000	.975	-0.003	.044	0.002	0.0210
		Strong	129.557	41	0.000	28.376	6	0.000	.979	0.006	.972	0.003	.047	-0.003	0.0313
		Strict	205.692	51	0.000	76.135	10	0.000	.964	0.015	.960	0.012	.055	-0.008	0.0220

χ^2: chi-square; df: degrees of freedom; CFI: comparative fit index; TLI: Tucker–Lewis index; RMSEA: root mean square error of approximation; CI: confidence interval; SRMR: standardized root mean residual.
