# Supplementary material for: The Depression Anxiety Stress Scale 21: Development and Validation of the Depression Anxiety Stress Scale 8-Item in Psychiatric Patients and the General Public for Easier Mental Health Measurement in a Post COVID-19 World
Source: Int J Environ Res Public Health. 2021 Sep 27;18(19):10142. doi: 10.3390/ijerph181910142 (PMC8507889; doi:10.3390/ijerph181910142)
Supplement: Supplementary file 1 [file ijerph-18-10142-s001.zip › Supplementary Table S2.pdf]

Supplementary Table S2. Invariance of factor structures of the shortened versions of the Depression Anxiety Stress Scale 21 across age groups

**Model**	**Groups**	**Invariance levels**	**χ^2**	**df**	***p***	**$\Delta\chi^2$**	**Δdf**	***p*($\Delta\chi^2$)**	**CFI**	**ΔCFI**	**TLI**	**ΔTLI**	**RMSEA**	**ΔRMSEA**	**SRMR**
3F 12-item	Sample 1	Configural	189.567	102	0.000				.923		.900		.072		0.0553
DASS		Metric	204.569	111	0.000	15.002	9	0.091	.917	0.006	.902	-0.002	.071	0.001	0.0679
		Strong	212.713	117	0.000	8.144	6	0.228	.915	0.002	.905	-0.003	.070	0.001	0.0688
		Strict	263.901	129	0.000	51.188	12	0.000	.881	**0.034**	.878	**0.027**	.079	-0.009	0.0756
3F 12-item	Sample 2	Configural	287.862	98	0.000				.942		.957		.044		0.0344
DASS		Metric	321.953	107	0.000	34.091	9	0.000	.940	0.002	.952	0.005	.045	10.001	0.0415
		Strong	390.435	113	0.000	68.482	6	0.000	.927	0.013	.937	0.015	.050	-0.005	0.0734
		Strict	754.881	127	0.000	364.447	14	0.000	.853	**0.074**	.858	**0.079**	.071	**-0.021**	0.0519
3F 13-item	Sample 1	Configural	226.640	124	0.000				.920		.899		.071		0.0578
DASS		Metric	240.351	134	0.000	13.711	10	0.187	.917	0.003	.903	-0.004	.069	0.002	0.0690
		Strong	243.335	140	0.000	2.984	6	0.811	.919	-0.002	.910	-0.007	.067	0.002	0.0689
		Strict	296.525	153	0.000	58.910	13	0.000	.888	**0.031**	.886	**0.024**	.075	**-0.008**	0.0765
3F 13-item	Sample 2	Configural	287.862	98	0.000				.957		.942		.044		0.0344
DASS		Metric	321.953	107	0.000	34.091	9	0.000	.952	0.005	.940	0.002	.045	-0.001	0.0415
		Strong	390.435	113	0.000	68.482	6	0.000	.937	0.015	.927	0.013	.050	-0.005	0.0734
		Strict	754.881	127	0.000	364.447	14	0.000	.858	**0.079**	.853	**0.074**	.071	**-0.021**	0.0519
Osman	Sample 1	Configural	76.225	46	.003				.955		.930		.063		0.0634
DASS-9		Metric	87.349	52	.002	11.124	6	0. 085	.948	0.007	.927	0.003	.064	-0.001	0.0735
		Strong	93.435	58	.002	6.086	6	0.414	.947	0.001	.935	-0.008	.061	0.003	0.0774
		Strict	136.920	68	.000	43.485	10	0.000	.898	**0.049**	.892	**0.043**	.078	**-0.017**	0.0870

Osman	Sample 2	Configural	114.610	44	0.000				0.973		.956		0.040		0.0275
DASS-9		Metric	117.188	50	0.000	2.578	6	0. 860	.974	-0.001	.963	-0.007	.037	0.003	0.0293
		Strong	208.313	56	0.000	91.124	6	0.000	.942	**0.032**	.925	**0.038**	.052	**-0.015**	0.0614
		Strict	474.624	67	0.000	266.311	11	0.000	.844	**0.098**	.832	**0.093**	.078	**-0.026**	0.0466
Our DASS-9	Sample 1	Configural	86.659	46	0.000				.965		.945		.073		0.0363
		Metric	91.846	52	0.001	5.187	6	0.520	.965	0.000	.952	-0.007	.068	0.005	0.0401
		Strong	100.156	58	0.000	8.311	6	0.216	.963	0.002	.955	-0.003	.066	-0.002	0.0438
		Strict	146.821	68	0.000	46.664	10	0.000	.932	**0.031**	.928	**0.027**	.084	**-0.018**	0.0582
Our DASS-9	Sample 2	Configural	182.671	46	0.000				.969		.952		.055		0.0301
		Metric	196.367	52	0.000	13.695	6	0.033	.968	0.001	.955	-0.003	.053	0.002	0.0348
		Strong	278.476	58	0.000	82.109	6	0.000	.951	0.017	.939	0.016	.062	-0.009	0.0690
		Strict	624.072	68	0.000	345.596	11	0.000	.875	**0.024**	.868	**0.029**	.091	**-0.029**	0.0530
Our DASS-8	Sample 1	Configural	58.341	32	.003				.974		.955		.070		0.0293
		Metric	63.981	37	.004	5.640	5	0.343	.974	0.00	.960	-0.005	.066	0.004	0.0350
		Strong	69.823	43	.006	5.842	6	0.441	.974	0.000	.966	-0.006	.061	0.005	0.0374
		Strict	115.584	52	.000	45.761	9	0.000	.938	**0.036**	.933	**0.033**	.086	**-0.025**	0.0551
Our DASS-8	Sample 2	Configural	104.693	30	0.000				.982		.967		.050		0.0227
		Metric	111.105	35	0.000	6.142	5	0.268	.982	0.000	.971	-0.004	.047	0.003	0.0251
		Strong	190.857	41	0.000	79.752	6	0.000	.964	0.018	.951	0.020	.061	0.014	0.0590
		Strict	531.167	51	0.000	340.309	10	0.000	.886	**0.078**	.874	**0.077**	.098	**-0.037**	0.0317

χ^2: chi-square; df: degrees of freedom; CFI: comparative fit index; TLI: Tucker–Lewis index; RMSEA: root mean square error of approximation; CI: confidence interval; SRMR: standardized root mean residual.
